# Tickborne disease awareness and protective practices among U.S. Forest Service employees from the upper Midwest, USA

**DOI:** 10.1186/s12889-020-09629-x

**Published:** 2020-10-20

**Authors:** Anna Schotthoefer, Kathryn Stinebaugh, Michael Martin, Claudia Munoz-Zanzi

**Affiliations:** 1 Marshfield Clinic Research Institute, 1000 N Oak Ave, Marshfield, WI 54449 USA; 2 Division of Epidemiology and Community Health, School of Public Health, University of Minnesota, Minneapolis, MN 55454 USA; 3 Chequamegon-Nicolet National Forest, Great Divide Ranger District, P. O. Box 896, Hayward, WI 54843 USA; 4 Division of Environmental Health Sciences, School of Public Health, University of Minnesota, Minneapolis, MN 55455 USA

**Keywords:** Lyme disease, Tick-borne disease, Occupational risk, Tick bites, Personal protective measures, Knowledge attitudes practices (KAP) survey

## Abstract

**Background:**

People with occupations that require them to spend time working outdoors in suitable tick habitats are predicted to be at an increased risk for tick-borne diseases (TBDs). However, few studies have assessed the risks of outdoor employees in the United States.

**Methods:**

We conducted a cross-sectional survey to collect data on exposure to ticks and TBD infections among U.S. Forest Service employees in a high TBD incidence region of northern Wisconsin, and to examine employee knowledge, attitudes, and practices (KAPs) regarding TBDs to help guide future education and prevention programs. Chi-square contingency tables, calculations of odds ratios, and logistic regression models were used to identify associations among self-reported employee factors, the proportion of correctly answered knowledge questions, their ranked concern for TBDs, adherence to practicing preventive behaviors, and willingness to pay for protective measures.

**Results:**

Ninety-five employees completed the survey. Nearly all respondents (97%) reported recent tick exposure, with 27% reporting encountering 10 or more ticks per week during peak tick season. Employee knowledge of TBD was high (median score: 80% correct). Fifty-nine percent of respondents had high concern for TBDs, and there was high adherence to conducting body checks for ticks (83% reported always doing them), but only moderate use of tick repellents (24% reported always and 60% reported occasionally using). High concern for TBD (adjusted odds ratio (aOR) = 6.32 [95% confidence intervals, 1.97–20.28]), a history of TBD diagnosis (aOR = 5.88 [1.41–24.55]), and older age (≥ 46 years) (aOR = 3.29 [1.00–10.84]) were positively associated with high practice adherence. Respondents suggested they would be willing to pay for personal protective methods and get a hypothetical vaccine for Lyme disease, but not community-wide efforts to control ticks.

**Conclusions:**

Our study provides evidence that U.S. Forest Service employees in Wisconsin represent a high risk group for TBD, and despite relatively high TBD knowledge and engagement in tick protection activities, efforts are needed to reduce their risks for tick bites. More generally, our findings suggest that studies to better understand the factors related to the adoption and effectiveness of public health interventions are needed.

## Background

Incidence of tick-borne diseases (TBD) are rising in North America, with Lyme disease (LD), caused by the bacterium *Borrelia burgdorferi*, currently ranking as one of the highest nationally notifiable diseases in the U.S. More than 30,000 annual confirmed or probable cases of the disease have been reported to the U.S. Centers for Disease Control and Prevention in recent years [1], with potentially 10 times more unreported cases occurring each year [2, 3]. LD often manifests as a red, expanding rash known as erythema migrans in 70–80% of cases [4, 5]. Other symptoms of the disease may include fever, arthralgia, myalgia, fatigue, malaise, and headache. If left untreated, infected individuals may develop late-stage symptoms affecting the musculoskeletal, neurologic, or cardiac systems [6, 7]. Persistent symptoms of fatigue, muscle and joint achiness, nonspecific neurologic symptoms, and the feeling of altered cognition following antibiotic treatment also have been recognized in some patients in a condition termed Post-Treatment Lyme disease Syndrome, though the causal mechanisms for the condition have not been identified [8]. With increasing incidence rates and the potential for long-term health impacts, the disease can have high personal and societal economic costs [9, 10].

Wisconsin is one of 14 high incidence LD states in the U.S., which account for about 95% of reported cases, and where the reported incidence is ≥10 annual cases per 100,000 people [5]. In these high incidence states, *B. burgdorferi* is spread by *Ixodes scapularis,* the black-legged or deer tick. The tick can transmit other pathogens that cause disease in humans, including the bacteria *Anaplasma phagocytophilum* and *Ehrlichia muris eauclairensis*, the parasite *Babesia microti,* and the Powassan virus [11]. Between 1996 and 2015, the number of counties reporting established *I. scapularis* populations in four of the upper Midwest states (Minnesota, Wisconsin, Michigan, and Illinois) expanded by 330%. For Wisconsin, the increase was 176% and it is believed that the tick is now established in all counties in the state with suitable habitat [12, 13]. Population densities of *I. scapularis* in the Midwest are positively associated with deciduous, dry to mesic forests, with well-draining soils [14, 15]; although, they may occur in many different types of habitats, including lawns [13, 16, 17]. In addition to *I. scapularis*, *Amblyomma americanum* (the lone star tick) populations are emerging in the state. This tick is responsible for the transmission of *Ehrlichia chaffeensis* [18].

Aside from living in or travelling to a high incidence region for TBDs, where the density of infected ticks may be high, individual-level factors associated with increased exposure to ticks and TBD pathogens are still poorly understood [19, 20]. Because of the increased amount of time spent in suitable tick habitats, it is believed that people in outdoor occupations may be at increased risk for TBD [21]. Seroprevalence studies suggest this may be particularly true for those who work in forestry [22–25]; however, risks associated with outdoor workers in the U.S. have not been adequately explored. Forests cover about 17 million acres, or approximately 46% of Wisconsin’s land cover, with about 9% of the forest in the Chequamegon-Nicolet National Forest (CNNF) located in the northern regions of the state where reported incidence rates for LD are highest [26, 27]. Anecdotal evidence provided to us suggested employees of CNNF have high tick exposure, and therefore, may be at increased risks for LD and other TBDs. To begin understanding the actual risks to this population of outdoor workers in Wisconsin, we conducted a cross-sectional survey to assess the knowledge, attitudes, and self-reported practices (KAP) regarding ticks and TBD. The results of this survey will help to identify opportunities for future education and initiatives to encourage workers with outdoor occupations to use effective measures to protect themselves from ticks and TBDs. Moreover, understanding how to improve adherence to protective measures in an at-risk group, may be applied more generally to public health efforts to encourage the adoption of TBD interventions.

## Methods

### Study participants

All 260 full- and part-time U.S. Forest Service employees of the CNNF in northern Wisconsin were eligible to participate in the study. An invitation to complete the KAP survey was disseminated to all employees by email in September 2016. Employees had been notified prior to the invitation that the survey study would be occurring. They had until the end of November 2016 to complete the survey, and two reminder emails were sent while the survey was open. The survey was administered online through Qualtrics [28], and the responses were stored anonymously. The study protocol and survey instrument were reviewed and approved by the Institutional Review Boards at the University of Minnesota and Marshfield Clinic Research Institute.

### Survey design

Questions were designed to capture participants’ knowledge of TBDs in terms of the disease life cycles and how they are transmitted to humans (Table S1). We used information as provided to the public, such as on the CDC and Wisconsin Division of Health websites [29, 30], to determine correct and incorrect answers to Knowledge questions. Questions also assessed participants’ attitudes regarding the potential implementation of various prevention methods (Table S2), including their willingness to pay for prevention methods, and their current use of personal protective practices (Table S3). Attitudes and practices associated with both personal and community-wide prevention methods were considered. Personal prevention methods include activities that are under individual control, such as use of tick repellents and conducting body checks for ticks, as well as making landscape modifications to reduce tick populations on personal properties. Community-wide prevention methods would be administered by local or regional community or municipality groups, and would include activities aimed at controlling tick populations, such as by broad-scale, environmental applications of acaricides, or reducing deer populations [31]. The survey instrument also collected demographic information, work history and environment, TBD history, and measures of potential tick exposure, including tick exposure at work and while doing other outdoor and recreational activities (Tables S4 and S5). Questions included True/False, multiple-choice, and free response style questions to measure KAP.

### Analysis of survey responses

Frequency distributions were used to summarize the characteristics of the respondent population and their answers to individual questions. We then created dichotomous variables by collapsing particular response categories or the Likert-scales used for questions to examine associations among respondent factors and outcomes that summarized KAPs, as described in the following sections. The respondent factors that we examined as potential explanatory variables in our analyses included sex (male, female), age (< 46 years, ≥ 46 years), length of employment in current position (≤ 10 years, > 10 years), number of hours working outdoors per week (≤ 20 h, > 20 h), reported history of at least one TBD diagnosis (yes, no), and estimates of tick exposure (low, high). For the latter, respondents that reported that they found ticks on themselves “always” or “most of the time” while working outdoors were categorized as reporting high tick exposure and respondents that reported finding ticks “sometimes,” or “never” were categorized as reporting low tick exposure at work.

The goal of our analysis was to explore the associations among respondent factors and our main outcomes of interest: TBD knowledge, concern, and practice adherence. In particular, we were interested in identifying potential factors associated with practice adherence, since this is ultimately what we would like to improve. We hypothesized that employees that reported a prior TBD diagnosis and high tick exposure would be more likely to have high knowledge, concern, and practice adherence. We also believed that working in positions that required more time spent outdoors, for more years would be potential modifiers of knowledge, concern, and practice adherence as these factors would likely be related to risks of tick exposure and TBD diagnosis. Following the exploratory analyses done in this study, we hope to propose and investigate casual models in future studies.

#### Knowledge

From the questions in this survey section (Table S1), a knowledge score was computed for each respondent using the True/False items, LD vector photo identification items, and the multiple-choice items on tick life stage and LD transmission. The scores were tallied with one point for each correct answer and zero points for incorrect answers for a total possible score of 15 points. Missing answers in this section of the survey (*n* = 4) were treated as incorrect and assigned zero points. Mean, median, and ranges for the scores were calculated and then knowledge scores were dichotomized such that participants with scores ≥ the median score were classified as having HIGH KNOWLEDGE, and participants with scores < the median were classified as having LOW KNOWLEDGE.

For the photo identification section, the question asked participants to select “yes” if they believed the arthropod was capable of transmitting LD to humans in Wisconsin/Minnesota, or “no” if they believed it was not. The images were presented to participants in random order.

#### Attitudes

We dichotomized the Likert-scale responses to questions that related to perceptions about TBD risk, LD vaccination, and willingness to pay for protection measures (Table S2). Participants that responded that TBDs are “very serious” were classified as having HIGH CONCERN, whereas participants that responded as “somewhat serious,” “not much of,” and “not a problem at all” were classified as LOW CONCERN. Likelihood of getting a LD vaccine, should one become available in the future, was also grouped into two categories: HIGH VACCINE if participants selected that “I would definitely get the vaccine” or “highly likely” and LOW VACCINE if they selected “maybe,” “unlikely,” or “I would not get the vaccine.” The willingness to pay for personal and community-based prevention measures was examined by collapsing responses into three categories. Respondents that selected “I am not willing to pay for any such efforts” were grouped as ZERO PAY, responders that selected that they would be willing to pay a maximum of “$5” or “$20”per year were grouped as TWENTY PAY, and respondents that selected a maximum of “$50,” “$100,” or “>$100” per year were grouped as FIFTY PAY.

#### Practices

Employees responded to a series of items asking how often they performed certain protective measures against ticks when working or doing recreational activities in tick habitats (Table S3). From the items that assessed the use of protective measures that are under individual control, a protective practice score was computed for each respondent. A score of zero was assigned for a “never” response, 0.5 for “occasionally”, and one for “always”; a total score of 7 points was possible (Table S3). A dichotomous practice outcome was assigned to each respondent as HIGH PRACTICE if the score was ≥ the median and LOW PRACTICE if the score was < the median.

### Statistical methods

Chi-square contingency tables and logistic regression models were used to analyze univariate associations among respondent factors and the dichotomized KAP outcome variables; specifically, we modeled the outcomes: HIGH KNOWLEDGE, HIGH CONCERN, and HIGH PRACTICE [32]. Additionally, we controlled for potential confounding among the respondent factors and each outcome in multivariate logistic regression models to obtain adjusted odds ratios for each factor. The 95% confidence intervals for the (a) odds ratios were used to evaluate significance in the tests and goodness-of-fit statistics were used to evaluate the models [32]. All analyses were performed using SAS 9.4.

## Results

Ninety-seven employees initiated the survey, however, two individuals only completed the demographic section and therefore were excluded; thus, we considered responses from 95 employees in our analysis, which corresponded to a 36.5% response rate. Sixty-five percent (61/95) of the respondents were male and 57% (54/95) were under 46 years old (18–25 years: 1%, 26–35 years: 25%, 36–45 years: 30%, and 46–65 years: 43%). The majority of respondents (74%, 70/95) had been in their current position for 10 years or more, and nearly all (98%, 93/95) reported that they worked outdoors, with 48% (46/95) working outdoors greater than 20 h per week (Table 1). Reported job titles included foresters, civil engineers or technicians, recreation technicians, administrators, and other positions related to biology, ecology, and natural resources.

**Table 1:** Work and tick exposure histories reported among U.S. Forest Service employees

Item	*n*	Response	Frequency (%)
Years in current job	95	< 1 year	5 (5)
		1 - < 5 years	8 (8)
		5–10 years	12 (13)
		> 10 years/My whole life	70 (74)
Required to work outdoors	95	No	5 (5)
		Yes	90 (95)
Hours/week working outdoors	95	Does not apply to me	2 (2)
		< 5 h	21 (22)
		5–10 h	15 (16)
		10–20 h	11 (12)
		> 20 h	46 (48)
Tick exposure during last 2 years	94	0 ticks	3 (3)
		< 10 ticks	20 (21)
		> 10 ticks	71 (76)
	71	If > 10 in last 2 years, how many during peak tick season?	
		1–5 ticks/week	35 (49)
		6–10 ticks/week	10 (14)
		> 10 ticks/week	25 (35)
		I cannot give a reliable answer	1 (1)
	71	If > 10 in last 2 years, where do you typically find them?	
		Work only	5 (7)
		Leisure/recreation only	1 (1)
		Both work and leisure/recreation	57 (80)
		Don’t know	8 (11)

Note: Variation in sample sizes for the first four items is due to non-responses by some respondents. The fifth and sixth items were only presented to those who reported seeing > 10 ticks on themselves in the last 2 years

### Tick exposure

Only three respondents reported that they had not found a tick on themselves within the last 2 years of the survey, whereas about 76% (71/94) of respondents reported they had “frequently” found ticks on themselves within that time period; 35% (25/71) of the latter reported finding 10 or more ticks per week during peak tick season. The majority of respondents that reported frequent tick exposure reported finding ticks on themselves both at work and during outdoor recreation activities (80%, 57/71) such as hiking, gardening, camping, and hunting (Table 1, Table S5). When asked about their tick exposure while engaging in specific activities, 70% of respondents (63/91) reported that they found ticks on themselves “always” or “most of the time” while working outdoors; these individuals were categorized as reporting high tick exposure at work. Of the remaining respondents, 26% reported finding ticks “sometimes,” and 3% reported “never” finding ticks while working outdoors and were categorized as reporting low tick exposure at work (Table S5).

### Disease history

Twenty-six percent (25/95) of respondents reported a history of TBD. All of these respondents reported they were diagnosed with LD by a medical provider, with 28% (7/25) of them reporting a history of LD and another TBD (Table 2). Forty-four percent (11/25) of the TBD respondents reported a single TBD diagnosis, whereas the remaining respondents reported either more than one separate diagnosis event (11/25), or possible diagnoses for coinfections (3/25). Among those who reported a history of at least one TBD diagnosis, 88% (22/25) reported that the diagnosis occurred while working at their current job, though our survey did not ask whether they thought infection was attributable to their work. Five percent of respondents (5/93) were currently being treated for a TBD at the time of the survey (Table 2).

**Table 2:** Reported tick-borne disease history among U.S. Forest Service employees

Item	*n*	Response	Frequency (%)
Ever diagnosed with a TBD by a medical provider?	95	Yes	25 (26)
		No	70 (74)
	25	If “Yes”, which diseases?^a^	
		Lyme disease	25 (100)
		Anaplasmosis	3 (3)
		Ehrlichiosis	5 (5)
		Babesiosis	2 (2)
		Other	1 (1)
	25	If “Yes”, about how many separate diagnoses?	
		1	14 (56)
		2	9 (36)
		3–4	0 (0)
		5+	2 (8)
	25	If “Yes,” did you receive this diagnosis while employed at your current occupation?	
		Yes	22 (88)
		No	3 (12)
Are you currently being treated for TBD?	93	Yes	5 (5)
		No	88 (95)

Note: Variation in sample sizes is due to non-responses by some respondents

^a^Respondents that answered “Yes” to the first item were presented with the list of diseases as shown and were able to select more than one; the Other response was a free response, for which the one respondent entered Bartonellosis. Of note in Wisconsin, anaplasmosis is still commonly called ehrlichiosis, as it was originally named [33], which may account for the higher number of reported cases of ehrlichiosis than anaplasmosis

A higher proportion of employees that had been at their job for more than 10 years reported a history of a TBD diagnosis (31% versus 12%; OR = 3.4 [0.91–12.43]); and, respondents that reported a high level of tick exposure at work were more likely to report a history of TBD diagnosis (35% versus 7%; OR = 6.7 [1.45–30.99]).

### Knowledge

**Fig. 1:**
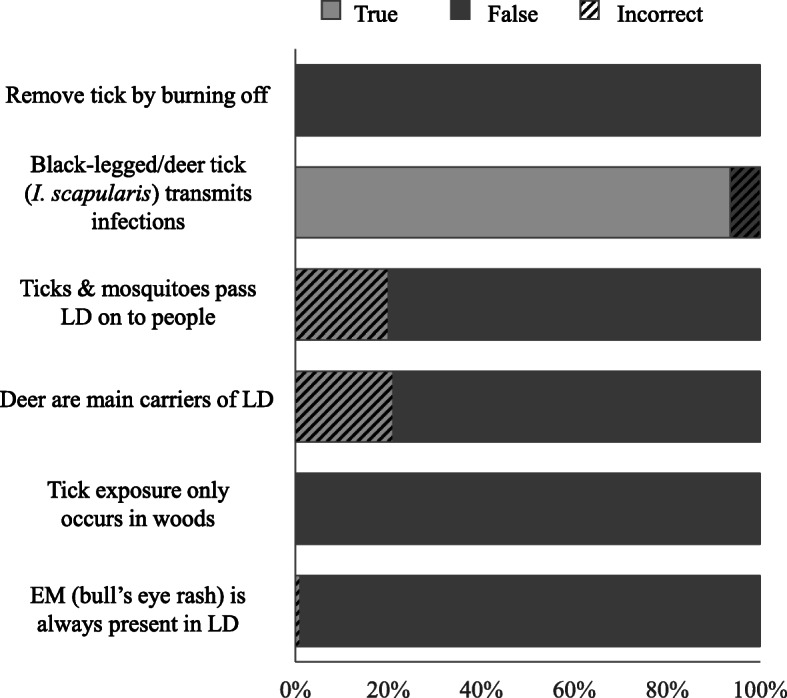
U.S. Forest Service employee responses to the True/False knowledge section of the survey (Table S1). All items were completed by 95 respondents except the second item, which was completed by 94 respondents

Sixty percent (57/95) of respondents correctly answered all True/False items (Fig. 1, Table S1). All respondents (95/95) correctly answered “False” to the statements “The best way to remove an attached tick is to burn it off” and “I only have to worry about tick exposure when I’m in the woods.” The most commonly missed items were “Both ticks and mosquitoes can pass Lyme disease on to people” and “Deer are the main carriers of Lyme disease” (Fig. 1). For the two multiple choice knowledge questions, the majority of respondents (59%, 56/95) correctly identified the nymph as the tick stage most likely to infect people with a TBD, whereas only 28% correctly selected summer as the season when ticks were most likely to transmit infections to humans; 66% incorrectly selected spring (Table S1).

**Fig. 2:**
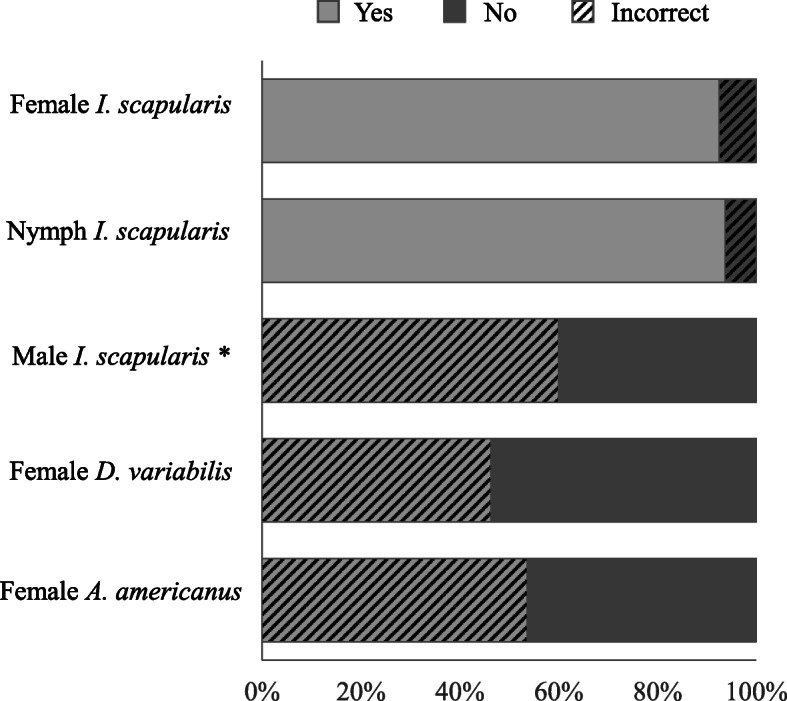
U.S. Forest Service employee responses to the LD vector identification section of the knowledge survey. Two other images not included here were an image of an adult bug (Hemiptera) and a spider; 95% of respondents correctly identified these latter two pictures as not being LD vectors. An answer was selected by 95 respondents for all, except the female *I. scapularis* and the spider pictures, which were answered by 94. * We considered “yes” as the incorrect answer for male *I. scapularis* because they are not known to feed for very long, and therefore, are not expected to transmit LD [34]; however, we are unaware of any experiments that have specifically examined transmission potential by males

Ninety-three percent (87/94) and 94% (89/95) of respondents correctly identified the photos of the adult female of *I. scapularis* and the nymph stage of *I. scapularis* as LD vectors, respectively (Fig. 2), but only 19% (18/94) correctly identified them exclusively. The most common incorrect responses included indicating that adult females of *A. americanus* (54%, 51/95) transmit LD. More than half of respondents (54%, 51/95) correctly identified that the adult female of *D. variabilis* is not a LD vector (Fig. 2).

The majority of participants (86%, 80/93) correctly selected the photo of the adult female *I. scapularis* as a LD vector and also responded “True” to the statement “The ticks most commonly associated with transmitting diseases to people in Wisconsin are known as black-legged ticks (also known as deer ticks; scientific name: *Ixodes scapularis*).” Seven percent (7/93) correctly identified the adult LD vector by name but not by photo, and 6% (6/93) correctly identified it by photo but not by name. No respondents missed both items. Of the 56 (59%) respondents who correctly answered the nymph life stage as the tick most likely to infect humans with a TBD, 95% (53/56) also selected the nymph by photo as a LD vector. Of the 38 (40%) respondents who incorrectly answered that the adult ticks were most likely to transmit a TBD to humans, 92% (35/38) still identified the nymph photo as a LD vector. Correctly, no respondents reported the larval stage ticks to be most likely to transmit TBDs to humans.

The computed knowledge scores (*n* = 95) had a mean of 11.6 (sd = 1.57), median of 12, and a range of 8–15. Sixty-eight percent (65/95) of respondents had a score ≥ median and were classified as HIGH KNOWLEDGE. The remaining 30 were classified as LOW KNOWLEDGE. Analyses did not reveal any statistically significant associations between knowledge level and sex, age category, years at job, hours per week worked outdoors, tick exposure, history of TBD diagnosis, TBD concern level, or practice adherence level (Table 3).

**Table 3:** Respondent characteristics, concern level and practice adherence status associated with scoring in the high knowledge category on the tick-borne diseases KAP survey

	*n*	High Knowledge, *n* (%)	Odds ratio (95% CI)	Adjusted OR (95% CI)
Sex				
Male	61	47 (77)	1.46 (0.56–3.78)	1.57 (0.57–4.36)
Female	33	23 (70)	1.0	
Age group				
≥ 46 years	41	31 (76)	1.09 (0.43–2.77)	1.17 (0.38–3.64)
< 46 years	54	40 (74)	1.0	
Years in current job				
> 10 years	70	53 (76)	1.21 (0.43–3.40)	1.16 (0.30–4.43)
≤ 10 years	25	18 (72)	1.0	
Time spent working outdoors				
> 20 h/week	46	34 (74)	0.92 (0.36–2.32)	0.94 (0.34–2.58)
≤ 20 h/week	49	37 (75)	1.0	
TBD diagnosis				
Yes	25	19 (76)	1.10 (0.38–3.17)	1.52 (0.45–5.09)
No	70	52 (74)	1.0	
Tick exposure				
High	71	52 (73)	0.58 (0.17–1.91)	0.52 (0.15–1.84)
Low	23	19 (83)	1.0	
Concern level				
High	56	44 (79)	1.63 (0.64–4.14)	1.79 (0.56–5.73)
Low	39	27 (69)	1.0	
Practice adherence				
High	61	44 (72)	0.67 (0.25–1.83)	0.41 (0.12–1.36)
Low	34	27 (79)	1.0	

Odds ratios (95% CIs) were estimated in univariate logistic regression models and aORs (95% CIs) were based on multivariate logistic regression models that included all of the respondent factors listed as explanatory variables

### Attitudes

For 59% (56/95) of the respondents, TBDs were of HIGH CONCERN. No one indicated TBDs were “not a problem at all.” When adjusting for all other respondent factors in the multivariate model for HIGH CONCERN, respondents in the older  age category (>= 46 years) were significantly less likely to be classified as HIGH CONCERN (aOR = 0.10 [0.03–0.41]), whereas respondents that reported working in their jobs for more than 10 years were more than 18 times more likely to be classified as HIGH CONCERN (aOR = 18.82 [4.11–86.17]; Table 4). Additionally, a positive association between TBD concern and adherence to protective measures was detected (aOR = 7.56 [2.20–26.05]; Table 4).

**Table 4:** Respondent characteristics, knowledge and practice adherence status associated with being classified as having high concern for tick-borne diseases

	*n*	High Concern, *n* (%)	Odds ratio (95% CI)	Adjusted OR (95% CI)
Sex				
Male	61	35 (57)	0.88 (0.37–2.07)	1.49 (0.49–4.51)
Female	33	20 (61)	1.0	
Age group				
≥ 46 years	41	21 (51)	0.57 (0.25–1.31)	0.10 (0.03–0.41)
< 46 years	54	35 (65)	1.0	
Years in current job				
> 10 years	70	48 (69)	4.64 (1.74–12.36)	18.82 (4.11–86.17)
≤ 10 years	25	8 (32)	1.0	
Time spent working outdoors				
> 20 h/week	46	25 (54)	0.69 (0.30–1.57)	0.68 (0.24–2.00)
≤ 20 h/week	49	31 (63)	1.0	
TBD diagnosis				
Yes	25	16 (64)	1.73 (0.52–3.43)	0.65 (0.18–2.33)
No	70	40 (57)	1.0	
Tick exposure				
High	71	42 (59)	0.93 (0.36–2.44)	0.49 (0.14–1.78)
Low	23	14 (61)	1.0	
Knowledge				
High	71	44 (62)	1.63 (0.64–4.14)	1.98 (0.61–6.36)
Low	24	12 (50)	1.0	
Practice adherence				
High	61	43 (70)	3.86 (1.59–9.34)	7.56 (2.20–26.05)
Low	34	13 (38)	1.0	

Odds ratios (95% CIs) were estimated in univariate logistic regression models and aORs (95% CIs) were based on multivariate logistic regression models that included all of the respondent factors listed as explanatory variables

When asked about their likelihood of getting a hypothetical LD vaccine, 75% of the respondents (70/93) selected that they would “definitely” or be “highly likely” to get one, placing them in the HIGH VACCINE category. Eighteen percent said they would “maybe” get a vaccine (17/93), and about 6% said they would be “unlikely” or that they “would not get the vaccine” (6/93), for the LOW VACCINE category. Employees who were < 46 years old had a greater odds of being classified in the HIGH VACCINE category compared with older employees (OR = 2.63 [1.00–6.93]).

**Fig. 3:**
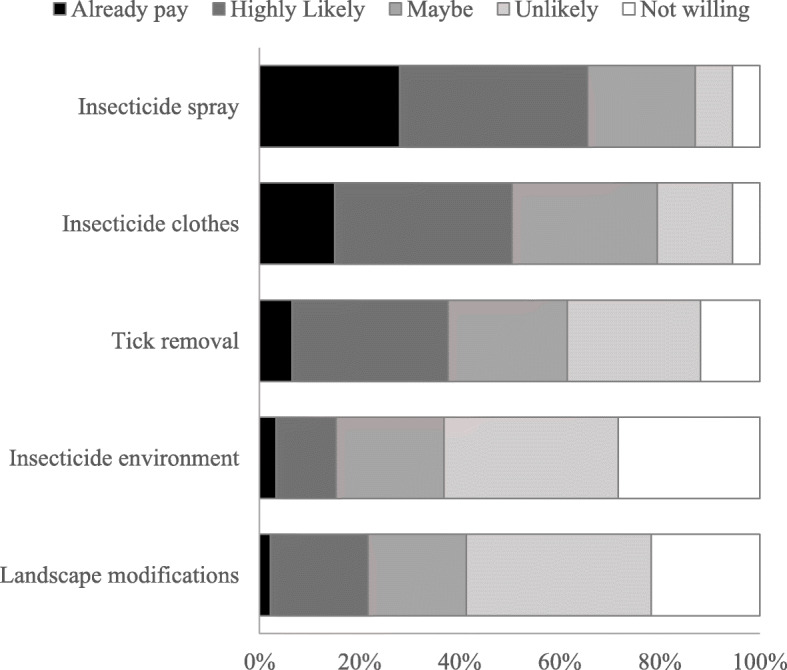
U.S. Forest Service employee responses to the willingness to pay for tick protection measures in the attitudes section of the survey. Participants responded to the question “Please rate your willingness to pay $20 or more per year for the following tick protection measures.” The sample size for the first three items was 93, and the sample size for the last two items was 92

In general, respondents indicated that they were willing to pay $20 per year for personal protective items, such as insecticides or protective clothing, but not for community-based interventions, such as applying insecticides to the environment or modifying habitats to control tick populations (Fig. 3). When given choices for the maximum amounts per year they would be willing to pay for personal tick control, the most frequent response was $50 (32%, 30/93). Twenty-five percent (23/93) reported a willingness to spend $100 or more per year; however, 12 respondents (13%) reported they would not be willing to pay any amount. By contrast, for community-level tick control efforts, the most popular response (49%, 45/92) was “not willing to pay any amount,” and 10% (9/92) reported they would be willing to pay $100 or more per year.

Women tended to be more likely to report that they would be willing to pay for personal protection items, with none of the women selecting that they would not be willing to pay for personal protections in contrast to 20% of the men, and 69% of women versus 50% of men selected that they would be willing to pay $50 or more each year (OR = 2.2 [0.89–5.43]). Respondents that selected maximum amounts of $50 or more for personal control items had a higher odds of being classified in the HIGH VACCINE category than respondents that selected amounts <$50 amounts (OR = 3.33 [1.14–10.44]).

### Practices

The computed practice scores (*n* = 94) had a mean of 3.57 (sd = 0.78), median of 3.5, and a range of 1.5–6.0. Sixty-four percent (60/94) of respondents were classified as HIGH PRACTICE. In addition to respondents that were classified as HIGH CONCERN, respondents that reported a TBD diagnosis were more likely to be classified in the HIGH PRACTICE category (aOR = 5.88 [1.41–24.55]; Table 5). Age also was a factor that influenced practice adherence, with respondents in the older age category (≥ 46 years old) having a slightly higher odds of being in the HIGH PRACTICE group (Table 5).

**Table 5:** Respondent characteristics, knowledge and concern level status associated with high practice adherence classification on the KAP survey

	*n*	High Practice, *n* (%)	Odds ratios (95% CI)	Adjusted OR (95% CI)
Sex				
Male	61	37 (61)	0.67 (0.27–1.65)	0.70 (0.24–2.07)
Female	33	23 (70)	1.0	
Age				
≥ 46 years	41	30 (73)	2.02 (0.84–4.86)	3.29 (1.00–10.84)
< 46 years	54	31 (57)	1.0	
Years in current job				
> 10 years	70	47 (67)	1.61 (0.63–4.09)	0.42 (0.11–1.58)
≤ 10 years	25	14 (56)	1.0	
Time spent working outdoors				
> 20 h/week	46	26 (56)	0.52 (0.22–1.22)	0.63 (0.23–1.71)
≤ 20 h/week	49	35 (71)	1.0	
TBD diagnosis				
Yes	25	21 (84)	3.94 (1.22–12.68)	5.88 (1.41–24.55)
No	70	40 (57)	1.0	
Tick exposure				
High	71	46 (65)	0.98 (0.37–2.63)	1.04 (0.32–3.35)
Low	23	15 (65)	1.0	
Knowledge				
High	71	44 (62)	0.67 (0.25–1.83)	0.40 (0.12–1.38)
Low	24	17 (71)	1.0	
Concern level				
High	56	43 (77)	3.86 (1.60–9.34)	6.32 (1.97–20.28)
Low	39	18 (46)	1.0	

Odds ratios (95% CIs) were estimated in univariate logistic regression models and aORs (95% CIs) were based on multivariate logistic regression models that included all of the respondent factors listed as explanatory variables

When we looked at factors related to the use of specific personal protective practices in univariate models, we found that being in the younger age group (< 46 years old) was positively associated with the use of acaricides (OR = 4.49 [1.31–15.39]), with no statistically significant differences in other practices by age. Being classified in the high tick exposure category tended to be positively associated with “always” conducting a body check for ticks (OR = 3.0 [0.97–9.33]), and although not statistically significant, respondents that reported a TBD diagnosis also tended to be more likely to report acaricide use (OR = 5.75 [0.71–46.30]).

**Fig. 4:**
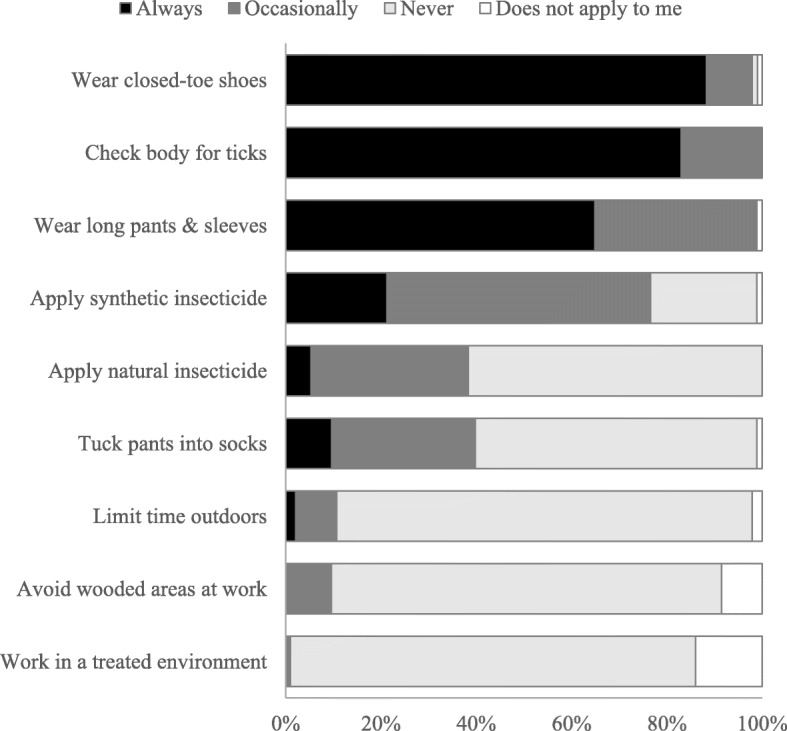
Tick protection practices reported by U.S. Forest Service employees. Participants responded to the question “Please provide information about the practices you may or may not engage in at work to protect yourself from ticks” Sample sizes for all items were 94, except for the fifth and ninth items, which were answered by 93 respondents

Most respondents (89%, 83/93) reported they always wear closed-toe shoes and more than half (66%, 61/93) reported they always wear long pants. About 24% and 60% of 93 respondents reported that they “always” or “occasionally” apply an acaricide (synthetic or natural), respectively, to their skin or clothing. The majority of respondents also reported that they “always” search their bodies for ticks after being outdoors at work (83%, 78/94), and no employees reported that they “never” do tick checks (Fig. 4). In contrast, nearly all respondents reported that they “never” work in an acaricide-treated environment (99%, 92/93), “never” avoid woody areas (90%, 85/94), and “never” limit their time outdoors (89%, 82/94) (Fig. 4).

## Discussion

Efforts to better understand where people are and the activities that they are engaged in when they are most likely to encounter infected ticks are needed to improve targeted interventions for controlling TBDs [19]. Although it is expected that people with outdoor occupations in high endemic areas will have an increased risk of TBD, studies to assess this assumption are largely lacking. In this study, we conducted a cross-sectional, KAP survey on U.S. Forest Service employees from a high incidence LD region. Our results corroborated anecdotal evidence that the employees in this region frequently come into contact with ticks, with nearly all respondents (91 of 94, 97%) acknowledging finding at least one tick on themselves within 2 years of the survey. In addition, respondents reported that frequent tick exposure was most commonly associated with working outdoors, rather than engaging in other outdoor activities for recreation or home maintenance. More than two-thirds of respondents indicated that they encounter ticks “always” or “most of the time” while working outdoors, whereas “sometimes” was most frequently selected for rating tick exposure during the other outdoor activities inquired about on the survey (Table S5). Respondents also reported a high rate of TBD; 25 (26%) reported a history of LD, including 7 individuals who also reported a diagnosis with at least one other TBD and 14 that reported more than one LD diagnoses or TBD illness events. Reporting high tick exposure while at work and working in their job for 10 years or more were positively associated with reporting a history of TBD. Although not directly comparable, these findings were consistent with other studies that detected a high seroprevalence of LD in forestry workers in Europe [22, 35].

Reports of high tick exposure and LD diagnoses in U.S. Forest Service employees in Wisconsin are concerning given the other results from our survey that suggested the employees have high knowledge, concern, and practice adherence for tick protection practices and TBD, which would be expected to reduce risks to tick bites. Over half of the respondents (60%) correctly answered all the knowledge True/False items, and 65% had a computed knowledge score that corresponded to 80% or more of all knowledge questions answered correctly. Though not directly comparable to KAP surveys conducted on other populations, these results were generally higher than those reported for other groups. For instance, Valente et al. [36] reported an average percentage of 43.5% of correct knowledge questions on a survey conducted on residents and visitors to Martha’s Vineyard, a highly LD endemic island off the coast of Massachusetts, and the majority of immigrant outdoor workers surveyed on the island reported that they had never heard of the disease or that they were not certain they could recognize the symptoms [37]. Employees of CNNF do receive some base level of tick and TBD education in the form of a presentation provided during safety meetings and they are provided with a tick identification card and Tick Fact Sheet that includes information about reporting tick bites, which may partially explain the high level of knowledge detected in our survey.

Personal protective measures that are recommended to prevent tick bites include wearing light-colored, long-sleeved shirts and long pants, tucking pant legs into socks, using a tick repellent on skin or clothing, and conducting checks for ticks following activities outdoors [8, 38]. All respondents reported engaging in one or more of these prevention practices. In particular, the majority of respondents reported always wearing pants, closed-toe shoes, and conducting body searches for ticks after working outdoors. Although most respondents (84%) indicated that they sprayed themselves or their clothing with an acaricide prior to working outdoors at least some of the time, only 24% reported that they always used an acaricide. Other TBD KAP surveys have found similar results with tick checks having a generally higher adherence than the use of tick repellents [39, 40]. Though our survey did not specifically address reasons for non-compliance, respondents reported using synthetic acaricides more frequently than natural acaricides (77% versus 38%), and only 7 individuals (7%) reported that they would use natural acaricides, but never used synthetic products, suggesting that non-adherence to always using an acaricide may not primarily be due to not trusting or wanting to use chemicals on their bodies or clothes.

We found that age and gender generally were not factors that influenced our respondents’ KAP toward TBD, though specific practices and attitudes did appear to be related to these factors. The younger age group, < 46 years old, was more likely to report using acaricides (92% versus 73%), whereas respondents older than 46 reported tucking their socks into their shoes more frequently (56% versus 28%). The younger age group also were more likely to state that they would definitely or be highly likely to get a LD vaccine (83% versus 65%). These results are encouraging for the younger age group; however, since LD is reported to be more common in older people [5], future efforts should attempt to better understand the perceived risks and benefits associated with specific practices in different age groups.

In addition to personal protection practices that are recommended to help prevent or reduce the likelihood of TBD, broader-scale changes to the environment that help to control tick populations or reduce the natural transmission of TBD pathogens are recommended to homeowners for reducing the risks of TBDs on their properties [41]. Such changes are also being explored as measures that may be applied on a community-wide scale to reduce the public health threats of TBDs. These measures include, broadly applying acaricides to the environment, removing shrubs or understory vegetation to reduce the suitability of habitats for ticks and reservoir hosts, or treating deer or mice with acaricides [31, 41]. Few respondents in our survey reported using insecticides in the environment to reduce tick populations or avoiding certain habitats to reduce their risks of tick exposure, as might be expected given that the nature of their work probably would not permit these actions; however, willingness to pay for such environmental control measures also was generally low. It is unclear if the latter is indicative of an unawareness that such measures have had some success at reducing tick populations [41, 42], or to more general perceptions that alterations to the environment are unacceptable [43]. Although studies are still needed to demonstrate the effectiveness of environmental control efforts for reducing tick populations or TBDs; e.g., [44], a better understanding of the perceived acceptability of such efforts may also be needed to identify those that are likely to have a high uptake by people at risk [43].

Despite demonstrating a moderate to high level of knowledge about LD, our survey detected one area that may be improved among employees with continued education. Although most respondents generally were able to visually differentiate ticks from other arthropods, few were able to identify photographs of *I. scapularis* ticks exclusively from other species of ticks: 19% selected only female and nymph *I. scapularis* as vectors of LD, and 27% selected female, nymph, and male *I. scapularis* only. In fact, following the latter, the most common combination of selected answers for the photograph section was answering “yes” to all of the photographs of ticks, regardless of species, and “no” to the two images of other arthropods (27% of respondents). Given that most respondents demonstrated an understanding that *I. scapularis*, or blacklegged ticks, were the vectors for LD in the True/False and multiple choice knowledge sections, failure to select them exclusively from the photographs displayed in the survey suggests that efforts to educate employees on the physical characteristics of *I. scapularis* ticks and how to differentiate them from other tick species may be needed. Although reducing exposure to all tick species may be a reasonable public health goal, having the knowledge to identify different species of ticks would be beneficial for employees because they may then be better equipped to assess their own personal risks for TBD after encountering or finding an attached tick. *Ixodes scapularis* and *A. americanum* ticks are known vectors of TBDs, whereas *D. variabilis* ticks are commonly encountered in Wisconsin, but are not known to transmit infections to humans. If there is an assumption that people use personal protective measures in response to their perceived risks of TBDs [40], as influenced by their personal experiences of picking up ticks, we think it would be important to know if their perceived risk is associated with encountering *I. scapularis* specifically or any ticks. Tick identification cards are commonly provided by public health and other agencies interested in educating the public about TBDs. If tick identification is viewed as a tool that may be used to help control TBDs, then evaluation of how the ability to identify ticks corresponds to adoption of personal protection measures should be investigated. Moreover, the ability to correctly distinguish species from each other would improve the reliability of any studies that are designed to specifically measure self-reported *I. scapularis* tick exposure rates. As such, in our study, although employees reported a high rate of encountering ticks, we are unable to know which species of ticks employees most frequently encounter.

Of interest from our survey also was that about two-thirds of respondents selected spring (March—May) as the season when ticks are most likely to transmit infections to humans. We determined this answer to be incorrect because summer (June—August) is the time of year associated with the highest infection rates in Wisconsin [30], and is generally reported as the riskest time of year on public health websites [29, 30]. Following administration of the survey, we learned that employees more frequently report encountering ticks in the spring compared to the summer and fall (M. Martin, personal communication); however, it is unclear which stage, or species, of ticks employees are frequently encountering in the spring to fully understand the response to the question. The life cycle of *I. scapularis* is such that the adults may be the stage of tick most active in the early spring (March—May) [45]. If employees are frequently encountering adults in the spring, their perceptions of infection risk may be influenced by the fact that adults are easier to detect than nymphs. The nymph is the stage of *I. scapularis* most likely to transmit infections to humans because of their smaller size and greater likelihood of going undetected, and they tend to become active in late spring, which may include late May [45, 46]. Employees may have selected spring because that answer included the month of May. Moreover, it is possible that employees are encountering nymphs earlier in the spring than the general population, as most outdoor activity for the general population may occur later in the year when weather improves (June–August). Further investigations are needed to fully understand the seasonal risks to people that work outdoors and if these risks may differ from the general public.

Limitations of our study include a fairly low response rate with the possibility that our results were biased towards those employees that were most interested in or concerned about TBDs. Though all employees were invited to complete the survey while they were at work, completion of the survey was voluntary and required employees to take their own initiative to complete it. Any future efforts to learn more about TBD KAPs among U.S. Forest Service employees may consider using more direct methods of engagement with employees, such as point-of-contact interviews, offering a small incentive to employees for completing surveys, or allowing for a longer window of time to complete the survey to improve the response rates. The self-reporting nature of the survey also does not allow for independent validation of the respondents’ answers, especially with regards to TBD diagnosis history. Despite these limitations, our results were consistent with previous reports of high tick and TBD exposure from the employees. Furthermore, the associations that we detected among the outcomes examined were consistent with the Health Belief Model (HBM), which has been used as the basis for other TBD KAP surveys [37, 40]. This model predicts that those that have a high perceived risk or have personal experience with a threat, respond best to education and tend to be more likely to adhere to protective practices. The U.S. Forest Service employees we surveyed had high exposure to ticks while working, and therefore, as would be expected based on the HBM had an overall high level of knowledge and concern about TBDs, as well as high adherence to personal protection practices. However, we did not question employees directly on their beliefs with regards to the efficacy of various protection practices, or their perceived barriers to using them, and therefore, additional research is needed to better understand the factors that were important in employee decisions to use or not use protection practices. Another limitation of our study was that it was not designed to determine if the employees are at a higher risk for TBD because of their occupation compared to others that do not work outdoors; therefore, though employees suggested that they more frequently encountered ticks while working compared to engaging in other outdoor activities, efforts are needed to measure risks associated with specific activities and where they occur to specifically define occupational versus recreational and peridomestic (e.g., yard work and gardening) risks associated with TBDs. For instance, the reported rate of LD diagnosis in our study population was similar to those reported in a recent survey of the general population in Connecticut and Maryland, two other high endemic states in the U.S. [39], such that it is unclear if the U.S. Forest Service employees that responded to our survey were at greater risk than the general population in our study region of northern Wisconsin, as the risk in the region is predicted to be high [47, 48]. Such knowledge would improve the allocation of limited public health resources to help prevent TBD infections.

## Conclusions

Incidence of LD and other TBDs have continued to rise and expand geographically. Though living or working in habitats where densities of infected ticks are high are known risk factors, there is a need for a better understanding of where and how humans come into contact with infected ticks and the factors that relate to adherence to personal protective practices. Understanding occupational risks and how to most effectively apply interventions to protect people while they are working outdoors, in particular, are needed. Our study supported assumptions that people spending long hours working outdoors in high risk areas frequently encounter ticks and may have a high risk for TBD. Though efforts to educate employees about ticks and TBD appear to be fairly successful, further training may be needed in the areas of tick identification and disease transmission processes. Wearing protective clothing and performing tick checks had high compliance, though application of tick repellents was reported to be less consistently used. Most employees were concerned about TBD and reported a willingness to pay for products that confer personal protection from ticks suggesting that intervention efforts targeting improved uptake of such practices would be beneficial.

## Supplementary information

**Additional file 1. **The survey instrument used in the study to assess tick-borne disease Knowledge, Attitudes, and Practices (KAP) in U. S. Forest Service employees, and summaries of reported outdoor activities and tick exposures while doing outdoor activities. **Table S1.** Knowledge survey. The questions and correct responses used to assess employee tick-borne disease Knowledge in the survey. **Table S2.** Attitudes survey. The questions and possible responses used in the Attitudes section of the survey. **Table S3.** Practices survey. The questions and possible responses used to assess employee use of protective practices in the Practices section of the survey. **Table S4.** Summary of outdoor activities. Frequencies (%) of respondents that indicated that they had done the outdoor activities listed at least 3 times per year while in Wisconsin/Minnesota. **Table S5.** Summary of tick exposure. Frequencies (%) of respondents that indicated that they had high or low tick exposure or that they never encountered ticks while doing the listed outdoor activities.

## Data Availability

The datasets analysed during the current study are available upon request to corresponding author.

## Abbreviations

aOR: Adjusted odds ratio
CNNF: Chequamegon-Nicolet National Forest
HBM: Health Belief Model
KAP: Knowledge Attitudes and Practices survey
LD: Lyme disease
OR: Odds ratio
TBD: Tick-borne disease

## References

[1] Centers for Disease Control and Prevention (2019). National Notifiable Diseases Surveillance System. 2018 Annual tables of infectious disease data.

[2] Hinckley AF, Connally NP, Meek JI, Johnson BJ, Kemperman MM, Feldman KA (2014). Lyme disease testing by large commercial laboratories in the United States. Clin Infect Dis..

[3] Nelson CA, Saha S, Kugeler KJ, Delorey MJ, Shankar MB, Hinckley AF (2015). Incidence of clinician-diagnosed Lyme disease, United States, 2005-2010. Emerg Infect Dis..

[4] Steere AC, Sikand VK (2003). The presenting manifestations of Lyme disease and the outcomes of treatment. N Engl J Med..

[5] Schwartz AM, Hinckley AF, Mead PS, Hook SA, Kugeler KJ (2017). Surveillance for Lyme disease - United States, 2008-2015. MMWR Surveill Summ..

[6] Steere AC, Strle F, Wormser GP, Hu LT, Branda JA, Hovius JW (2016). Lyme borreliosis. Nat Rev Dis Primers..

[7] Bush LM, Vazquez-Pertejo MT (2018). Tick borne illness-Lyme disease. Dis Mon..

[8] Wormser GP, Dattwyler RJ, Shapiro ED, Halperin JJ, Steere AC, Klempner MS (2006). The clinical assessment, treatment, and prevention of Lyme disease, human granulocytic anaplasmosis, and babesiosis: clinical practice guidelines by the Infectious Diseases Society of America. Clin Infect Dis..

[9] Adrion ER, Aucott J, Lemke KW, Weiner JP (2015). Health care costs, utilization and patterns of care following Lyme disease. PLoS One..

[10] Mac S, da Silva SR, Sander B (2019). The economic burden of Lyme disease and the cost-effectiveness of Lyme disease interventions: a scoping review. PLoS One..

[11] Nelder MP, Russell CB, Sheehan NJ, Sander B, Moore S, Li Y (2016). Human pathogens associated with the blacklegged tick *Ixodes scapularis*: a systematic review. Parasit Vectors..

[12] Eisen RJ, Eisen L, Beard CB (2016). County-scale distribution of *Ixodes scapularis* and *Ixodes pacificus* (Acari: Ixodidae) in the continental United States. J Med Entomol..

[13] University of Wisconsin-Madison, Department of Entomology. Wisconsin ticks and tick-borne diseases. https://wisconsin-ticks.russell.wisc.edu. Accessed 22 Sept 2020.

[14] Guerra M, Walker E, Jones C, Paskewitz S, Cortinas MR, Stancil A (2002). Predicting the risk of Lyme disease: habitat suitability for *Ixodes scapularis* in the north Central United States. Emerg Infect Dis..

[15] Johnson TL, Bjork JK, Neitzel DF, Dorr FM, Schiffman EK, Eisen RJ (2016). Habitat suitability model for the distribution of *Ixodes scapularis* (Acari: Ixodidae) in Minnesota. J Med Entomol..

[16] Rydzewski J, Mateus-Pinilla N, Warner RE, Hamer S, Weng HY (2011). *Ixodes scapularis* and *Borrelia burgdorferi* among diverse habitats within a natural area in east-Central Illinois. Vector Borne Zoonotic Dis..

[17] Coyle DD, Murphy MW, Paskewitz SM, Orrock JL, Lee X, Murphy RJ (2013). Belowground herbivory in red pine stands initiates a cascade that increases abundance of Lyme disease vectors. For Ecol Manag..

[18] Christenson M, Lee X, Larson S, Johnson DH, Jensen J, Meller M (2017). Occurrence of *Amblyomma americanum* (Acari: Ixodidae) and human infection with *Ehrlichia chaffeensis* in Wisconsin, 2008-2015. J Med Entomol..

[19] Eisen L, Eisen RJ (2016). Critical evaluation of the linkage between tick-based risk measures and the occurrence of Lyme disease cases. J Med Entomol..

[20] Connally NP, Durante AJ, Yousey-Hindes KM, Meek JI, Nelson RS, Heimer R (2009). Peridomestic Lyme disease prevention: results of a population-based case-control study. Am J Prev Med.

[21] Piacentino JD, Schwartz BS (2002). Occupational risk of Lyme disease: an epidemiological review. Occup Environ Med..

[22] Cisak E, Zajac V, Wojcik-Fatla A, Dutkiewicz J (2012). Risk of tick-borne diseases in various categories of employment among forestry workers in eastern Poland. Ann Agric Environ Med..

[23] Di Renzi S, Martini A, Binazzi A, Marinaccio A, Vonesch N, D'Amico W (2010). Risk of acquiring tick-borne infections in forestry workers from Lazio, Italy. Eur J Clin Microbiol Infect Dis..

[24] Rigaud E, Jaulhac B, Garcia-Bonnet N, Hunfeld KP, Femenia F, Huet D (2016). Seroprevalence of seven pathogens transmitted by the Ixodes ricinus tick in forestry workers in France. Clin Microbiol Infect.

[25] Wallace JW, Nicholson WL, Perniciaro JL, Vaughn MF, Funkhouser S, Juliano JJ (2016). Incident tick-borne infections in a cohort of North Carolina outdoor workers. Vector Borne Zoonotic Dis..

[26] Rogers ER, Christenson M, Sanderson S, Schumacher P, Jensen J, Hoang Johnson D (2016). Lyme disease trends in Wisconsin: Wisconsin environmental public health tracking program.

[27] University of Wisconsin Stevens Point, Center for Land Use Education (2019). The Wisconsin Land Use Megatrends series: Forests.

[28] Qualtrics. https://www.qualtrics.com. Accessed 22 Sept 2020.

[29] Centers for Disease Control and Prevention (2019). Lyme disease.

[30] Wisconsin Department of Health Services (2018). Lyme disease.

[31] Eisen L, Dolan MC (2016). Evidence for personal protective measures to reduce human contact with blacklegged ticks and for environmentally based control methods to suppress host-seeking blacklegged ticks and reduce infection with Lyme disease spirochetes in tick vectors and rodent reservoirs. J Med Entomol..

[32] Stokes ME, Davis CS, Koch GG (2000). Categorical data analysis using the SAS system.

[33] Chen SM, Dumler JS, Bakken JS, Walker DH (1994). Identification of a granulocytotropic *Ehrlichia* species as the etiologic agent of human disease. J Clin Microbiol..

[34] Eisen L (2018). Pathogen transmission in relation to duration of attachment by *Ixodes scapularis* ticks. Ticks Tick Borne Dis..

[35] De Keukeleire M, Robert A, Luyasu V, Kabamba B, Vanwambeke SO (2018). Seroprevalence of *Borrelia burgdorferi* in Belgian forestry workers and associated risk factors. Parasit Vectors..

[36] Valente SL, Wemple D, Ramos S, Cashman SB, Savageau JA (2015). Preventive behaviors and knowledge of tick-borne illnesses: results of a survey from an endemic area. J Public Health Manag Pract..

[37] Heller JE, Benito-Garcia E, Maher NE, Chibnik LB, Maher CP, Shadick NA (2010). Behavioral and attitudes survey about Lyme disease among a Brazilian population in the endemic area of Martha's Vineyard, Massachusetts. J Immigr Minor Health..

[38] Piesman J, Eisen L (2008). Prevention of tick-borne diseases. Annu Rev Entomol..

[39] Beaujean DJ, Bults M, van Steenbergen JE, Voeten HA (2013). Study on public perceptions and protective behaviors regarding Lyme disease among the general public in the Netherlands: implications for prevention programs. BMC Public Health..

[40] Niesobecki S, Hansen A, Rutz H, Mehta S, Feldman K, Meek J (2019). Knowledge, attitudes, and behaviors regarding tick-borne disease prevention in endemic areas. Ticks Tick Borne Dis..

[41] Stafford KC 3rd. Tick Management Handbook: An integrated guide for homeowners, pest control operators, and public health officials for the prevention of tick-associated disease, revised edition: The Connecticut Agricultural Experiment Station; 2007. Bulletin No. 1010:80. Available at: https://stacks.cdc.gov/view/cdc/11444. Acessed 5 Oct 2020.

[42] Curran KL, Fish D, Piesman J (1993). Reduction of nymphal Ixodes dammini (Acari: Ixodidae) in a residential suburban landscape by area application of insecticides. J Med Entomol..

[43] Aenishaenslin C, Michel P, Ravel A, Gern L, Milord F, Waaub JP (2016). Acceptability of tick control interventions to prevent Lyme disease in Switzerland and Canada: a mixed-method study. BMC Public Health..

[44] Hinckley AF, Meek JI, Ray JA, Niesobecki SA, Connally NP, Feldman KA (2016). Effectiveness of residential Acaricides to prevent Lyme and other tickborne diseases in humans. J Infect Dis..

[45] Piesman J (1993). Dynamics of Borrelia burgdorferi transmission by nymphal Ixodes dammini ticks. J Infect Dis..

[46] Porter WT, Motyka PJ, Wachara J, Barrand ZA, Hmood Z, McLaughlin M (2019). Citizen science informs human-tick exposure in the northeastern United States. Int J Health Geogr..

[47] Diuk-Wasser MA, Hoen AG, Cislo P, Brinkerhoff R, Hamer SA, Rowland M (2012). Human risk of infection with Borrelia burgdorferi, the Lyme disease agent, in eastern United States. Am J Trop Med Hyg..

[48] Knox KK, Thomm AM, Harrington YA, Ketter E, Patitucci JM, Carrigan DR (2017). Powassan/deer tick virus and Borrelia Burgdorferi infection in Wisconsin tick populations. Vector Borne Zoonotic Dis..

