# Supplementary material for: Tickborne disease awareness and protective practices among U.S. Forest Service employees from the upper Midwest, USA
Source: BMC Public Health. 2020 Oct 20;20:1575. doi: 10.1186/s12889-020-09629-x (PMC7574197; doi:10.1186/s12889-020-09629-x)
Supplement: Supplementary file 1 — Additional file 1. The survey instrument used in the study to assess tick-borne disease Knowledge, Attitudes, and Practices (KAP) in U. S. Forest Service employees, and summaries of reported outdoor activities and tick exposures while doing outdoor activities. Table S1. Knowledge survey. The questions and correct responses used to assess employee tick-borne disease Knowledge in the survey. Table S2. Attitudes survey. The questions and possible responses used in the Attitudes section of the survey. Table S3. Practices survey. The questions and possible responses used to assess employee use of protective practices in the Practices section of the survey. Table S4. Summary of outdoor activities. Frequencies (%) of respondents that indicated that they had done the outdoor activities listed at least 3 times per year while in Wisconsin/Minnesota. Table S5. Summary of tick exposure. Frequencies (%) of respondents that indicated that they had high or low tick exposure or that they never encountered ticks while doing the listed outdoor activities. [file 12889_2020_9629_MOESM1_ESM.docx]

**Additional file 1.**

Table S1: Knowledge survey. The questions and correct responses used to assess employee tick-borne disease Knowledge in the survey.

*Knowledge Questions*

|  | Item | Responses |
| --- | --- | --- |
| 1. | The best way to remove an attached tick is to burn it off | True  **False** |
| 2. | The ticks most commonly associated with transmitting diseases to people in WI/MN are known as black-legged ticks (also known as deer ticks; scientific name *Ixodes scapularis*) | **True**  False |
| 3. | Both ticks and mosquitos can pass Lyme disease on to people | True  **False** |
| 4. | Deer are the main carriers of Lyme disease | True  **False** |
| 5. | I only have to worry about tick exposure when I’m in the woods | True  **False** |
| 6. | 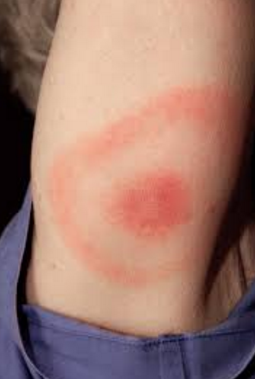Erythema migrans (bullseye rash – pictured below) is always present with Lyme disease | True  **False** |
| 7. | Which life stage of the tick is most likely to infect humans with a tick-borne disease? | Larva  **Nymph**  Adult |
| 8. | The time of year when ticks are most likely to transmit infections to humans is? | Spring (Mar-May)  **Summer (Jun-Aug)**  Fall (Sep-Oct) |
| 9-15. | Please select “YES” if you believe the arthropod represented in the picture is capable of transmitting Lyme disease to humans in Wisconsin/Minnesota, or “NO” if you believe it is not. | *See Results section Figure 3 for photos and answers* |

*Note.* Correct responses are shown in bold. All item responses were used to calculate the knowledge score for each participant.

Table S2: Attitudes survey. The questions and possible responses used in the Attitudes section of the survey.

*Attitude Questions*

|  | Item | Responses |
| --- | --- | --- |
| 1. | What is your current level of concern regarding tick-borne diseases? | Not a problem at all  Not much of  Somewhat serious  Very Serious |
|  | Please rate your willingness to pay $20 or more per year for the following tick protection measures: |  |
| 2. | Insecticides that I can apply to my skin, clothing or work area to protect myself from ticks | I am not willing to pay  Unlikely  Maybe  Highly likely  I do pay this amount already |
| 3. | Clothing that comes with insecticides embedded in the fabric | *As in 2.* |
| 4. | Tick removal equipment (such as tweezers, magnifying glasses, etc.) | *As in 2.* |
| 5. | Application of insecticides to workplace to control ticks | *As in 2.* |
| 6. | Landscape modifications that would reduce the amount of tick habitat in your workplace | *As in 2.* |
| 7. | What is the maximum amount per year that you would be willing to pay for personal tick control efforts, such as insecticide spray or tick removal equipment (such as tweezers, magnifying glasses, etc.)? | I am not willing to pay for such efforts  $5  $20  $50  $100  > $100 |
| 8. | What is the maximum amount per year that you would be willing to pay for community-based tick control efforts? | *As in 7.* |
| 9. | If a safe, effective vaccine was developed for Lyme disease, how willing would you be to getting vaccinated? | I would not get the vaccine  Unlikely  Maybe  Highly Likely  I would definitely get the vaccine |
|  | Regarding previous question, why or why not would you be willing to be vaccinated for Lyme disease? | *Free response* |

*Note.* Responses to the numbered questions were transformed into dichotomized or three category response variables in analyses.

Table S3: Practices survey. The questions and possible responses used to assess employee use of protective practices in the Practices section of the survey.

*Practice Questions*

|  | Item | Responses |
| --- | --- | --- |
|  | If you had a tick attached to your skin, did you attempt to remove it? If so, how? | Yes, removed/squeezed with fingers  Yes, burned with a match/lighter  Yes, twisted out with tweezers  Yes, pulled straight out with tweezers  Yes, applied a substance (for example – Vaseline or rubbing alcohol)  No, I had a tick attached to my skin but I did not attempt to remove it  No, I have never found a tick on myself |
|  | Do you follow your employer’s policy for reporting tick exposures? | Always  Sometimes  Never  I do not know my employer’s policy for reporting tick exposure |
|  | Please provide information about the practices you may or may not engage in at work to protect yourself from ticks: |  |
| 1. | I use or wear clothing treated with a synthetic insecticide, such as DEET or permethrin (i.e. - InsectShield) | Does not apply to me  Never  Occasionally  Always |
| 1. | I spray myself or my clothes with a natural insecticide, such as lemon-scented eucalyptus oil, lavender oil, or Alaska yellow cedar oil | *As in 1.* |
| 2. | I wear long-sleeved shirts and pants | *As in 1.* |
| 3. | I wear closed-toe shoes | *As in 1.* |
| 4. | I tuck my pants into my socks | *As in 1.* |
| 5. | I search my body for ticks after being outdoors | *As in 1.* |
|  | The environment in my workplace is sprayed with insecticides that are effective against ticks (e.g. permethrin, cyfluthrin, deltamethrin) |  |
| 6. | I avoid woody areas during work | *As in 1.* |
| 7. | I limit my time spent outdoors when possible | *As in 1.* |

*Note.* Responses to the numbered questions were used to calculate the practice adherence scores for each participant.

Table S4. Summary of outdoor activities. Frequencies (%) of respondents that indicated that they had done the outdoor activities listed at least 3 times per year while in Wisconsin/Minnesota.

| **Outdoor activity** | **Frequency (%) that affirmed doing ≥ 3 times per year** |
| --- | --- |
| Hiking | 85 (89%) |
| Camping | 55 (58) |
| Hunting | 53 (56) |
| Gardening | 58 (61) |
| Yard landscaping | 75 (79) |
| Bird watching | 28 (29) |

Table S5: Summary of tick exposure. Frequencies (%) of respondents that indicated that they had high or low tick exposure or that they never encountered ticks while doing the listed outdoor activities.

| **Outdoor activity** | **Number that answered *** | **High (%)** | **Low (%)** | **Never (%)** |
| --- | --- | --- | --- | --- |
| Camping | 82 | 17 (21) | 47 (57) | 9 (11) |
| Hiking | 88 | 42 (48) | 42 (48) | 2 (2) |
| Doing yard work | 87 | 19 (22) | 48 (55) | 19 (22) |
| Chopping wood | 83 | 11 (13) | 31 (37) | 25 (30) |
| Hunting in spring | 82 | 22 (27) | 20 (24) | 7 (8) |
| Hunting in fall | 82 | 14 (17) | 43 (52) | 6 (7) |
| Fishing from shore | 83 | 9 (11) | 33 (40) | 16 (19) |
| Gardening | 83 | 11 (13) | 25 (30) | 29 (35) |
| Bird watching | 83 | 12 (14) | 17 (20) | 16 (19) |
| Working outdoors | 91 | 63 (69) | 24 (26) | 3 (3) |

* If respondents indicated that they had had tick exposure within 2 years of the survey, they were then asked to rate their frequency of finding ticks on their clothing or attached to their bodies while engaging in specific activities, including working outdoors. We categorized responses as High if respondents selected “most of the time” or “always”, Low if “sometimes” was selected, and Never if “never” was selected. The total includes respondents that also selected the option, “I do not participate in specified activity.”
